# Aberrant Expression of Long Non Coding RNA HOTAIR and De-Regulation of the Paralogous 13 HOX Genes Are Strongly Associated with Aggressive Behavior of Gastro-Entero-Pancreatic Neuroendocrine Tumors

**DOI:** 10.3390/ijms22137049

**Published:** 2021-06-30

**Authors:** Annabella Di Mauro, Giosuè Scognamiglio, Gabriella Aquino, Margherita Cerrone, Giuseppina Liguori, Ottavia Clemente, Maurizio Di Bonito, Monica Cantile, Gerardo Botti, Salvatore Tafuto, Fabiana Tatangelo

**Affiliations:** 1 Pathology Unit, Istituto Nazionale Tumori Fondazione “G. Pascale”, Via Mariano Semmola, 80131 Naples, Italy; annabella.dimauro@istitutotumori.na.it (A.D.M.); giosue.scognamiglio@istitutotumori.na.it (G.S.); g.aquino@istitutotumori.na.it (G.A.); margherita.cerrone@istitutotumori.na.it (M.C.); g.liguori@istitutotumori.na.it (G.L.); m.dibonito@istitutotumori.na.it (M.D.B.); f.tatangelo@istitutotumori.na.it (F.T.); 2 Sarcomas and Rare Tumors Unit, Istituto Nazionale Tumori-IRCCS-Fondazione G. Pascale, Via Mariano Semmola, 80131 Naples, Italy; ottavia.clemente@istitutotumori.na.it (O.C.); s.tafuto@istitutotumori.na.it (S.T.); 3 Scientific Direction, Istituto Nazionale Tumori-IRCCS-Fondazione G. Pascale, Via Mariano Semmola, 80131 Naples, Italy; g.botti@istitutotumori.na.it

**Keywords:** GEP-NENs, HOX genes, lncRNA *HOTAIR*

## Abstract

Gastro-entero-pancreatic neuroendocrine neoplasms (GEP-NENs) are rare diseases occurring in the gastrointestinal tract and pancreas. They are characterized by the loss of epithelial tubular gland elements, and by the increased expression of neuroendocrine markers. GEP-NENs are subdivided into two histo-pathological types, gastro-entero-pancreatic neuroendocrine tumors (GEP-NETs) and gastro-entero-pancreatic neuroendocrine carcinomas (GEP-NECs). According to WHO 2017 and 2019 classification criteria are graded and staged in four categories, NET-G1, NET-G2, NET-G3, and NEC-G3. The molecular characterization of these tumors can be fundamental for the identification of new diagnostic, prognostic and predictive biomarkers. The main purpose of this study was to analyze the expression of the paralogous 13 HOX genes, normally involved in embryogenic development and frequently deregulated in human cancers, and of the HOX regulating lncRNA *HOTAIR* in GEP-NENs. The expression of HOX genes is gradually lost in the transition from GEP NET G1 to NET/NEC G3 tumors, while *HOTAIR* expression, inversely correlated with HOX genes expression and weakly expressed in low-grade GEP NENs, becomes aberrant in NET G3 and NEC G3 categories. Our data highlights their potential role in the molecular stratification of GEP-NENs by suggesting new prognostic markers and potential therapeutic targets.

## 1. Introduction

Neuroendocrine neoplasms are a heterogeneous group of rare tumors that can arise from different anatomic sites including foregut, midgut, hindgut, lung, bladder, prostate, adrenal gland and sympathetic nervous organs [1]. The term “neuro” is associated with the presence of dense core granules that are similar to those present in serotonergic neurons, which store monoamines, while the “endocrine” property refers to the synthesis and secretion of these monoamines [2]. Tumors arising from gastrointestinal tract, also defined Gastro-entero-pancreatic neuroendocrine tumors (GEP-NET) account for two-thirds of NENs.

The World Health Organization (WHO) 2017 (for Pancreatic neuroendocrine neoplasms [3] and 2019 (for gasto-enteric neuroendocrine neoplasms) [4] histopathological classification, introducing as classifying criteria tumor grading and cell differentiation, identified four categories for GEP-NEN tumors: (i) well-differentiated Neuroendocrine tumors, grade 1 (NET-G1); (ii) well-differentiated Neuroendocrine tumor, grade 2 (NET-G2); (iii) high proliferating well-differentiated Neuroendocrine tumor, grade 3 (NET-G3); (iv) poorly differentiated Neuroendocrine carcinoma, grade 3 (NEC-G3). The basis of the classification criteria also includes the integration of proliferation index Ki67 and identified: low grade NET G1 with Ki67 index 3%; intermediate grade NET G2 with Ki67 index >3–20%; intermediate grade NET G3 with Ki67 index >20–55%; high grade NEC G3 with Ki67 index: >55%.

All these categories are specific entities with different prognostic and therapeutic implications, for which a molecular characterization to identify new diagnostic and therapeutic tools, useful in their management, should be required [5].

HOX genes play a main role during the embryonic development, controlling the identity of different regions along the body axis, from the branchial area to the tail [6]. In cancer diseases, HOX genes are involved in different oncogenic processes, such as the control of cell differentiation, proliferation, apoptosis, cell invasion and epithelial–mesenchymal transition [7]. In particular, the genes belonging to HOX paralogous group 13 (*HOXA13*, *HOXB13*, *HOXC13*, and *HOXD13*) are strongly associated with cancer development and progression [8,9,10,11,12]. Posterior genes of the HOX genes network are involved in the development of the gut [13], and emerging evidences suggest that their de-regulation can be associated with exocrine tumor of pancreas [14] and colon cancers evolution [15].

HOX genes expression can be modulated by different non-coding RNAs (ncRNAs) some of which are localized within HOX loci [16]. In particular, the long non-coding RNA (lncRNA) HOTAIR (Hox transcript antisense intergenic RNA) is able to modulate metastatic processes in several human cancers [17].

Since the de-regulation posterior genes of the HOX genes network has been recently described also in some tumors with neuroendocrine differentiation [18], in this study we aimed to investigate the role of the paralogous group 13 HOX genes in a case series of GEP-NEN patients. We also evaluated the role of lncRNA *HOTAIR*, a regulator of posterior HOX genes expression, recently associated with GEP-NEN tumor progression [19].

## 2. Results

### 2.1. Clinic-Pathological Characteristics of GEP-NEN Patients

In our cohort, we have included 34 GEP-NEN patients, 50% women and 50% men. The age of patients ranged from 32–86 years, with an average age of 65 years. Regarding tumor location, 10/34 patients (29.4%) had a colon localization, 6/34 patients (17.6%) ileus, 5/34 patients (14.7%) pancreas and 13/34 cases (38.2%) were metastatic with a liver localization from GEP-NET. Classification based on tumor grading and differentiation identified 14/34 (41.2%) NET G1 patients, 7/34 (20.6%) NET G2, 3/34 (8.8%) NET G3 and 10/34 (29.4%) NEC G3. Clinic-pathological features are schematized in Table 1.

### 2.2. Paralogous 13 HOX Proteins Expression in GEP-NEN Patients in GEP-NEN Categories

**Figure 1:**
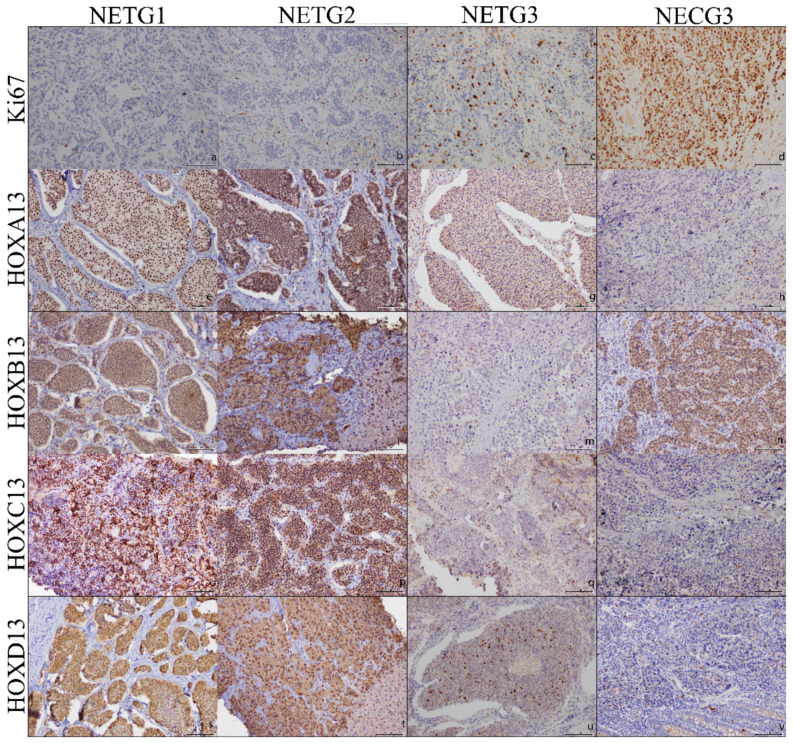
Paralogous 13 HOX proteins expression in GEP-NEN tumor categories: (**a**) NETG1 with Ki67 expression <3% (×20); (**b**) NETG2 with Ki67 expression between 3–20%; (**c**) NETG3 with Ki67 expression >20%; (**d**) NECG3 with Ki67 expression >20%; (**e**) positive *HOXA13* nuclear expression in NETG1 (×20); (**f**) positive *HOXA13* nuclear expression in NETG2 (×20); (**g**) negative *HOXA13* expression NETG3 (×20); (**h**) positive *HOXA13* nuclear expression in NECG3 (×20); (**i**) negative *HOXB13* in NETG1 (×20); (**l**) negative *HOXB13* in NETG2 (×20); (**m**) positive *HOXB13* cytoplasm expression NETG3 (×20); (**n**) positive *HOXB13* nuclear expression in NECG3 (×20); (**o**) positive *HOXC13* nuclear and cytoplasm expression in NETG1 (×20); (**p**) positive *HOXC13* nuclear expression in NETG2 (×20); (**q**) positive *HOXC13* nuclear expression in NETG3 (×20); (**r**) negative *HOXC13* expression in NEC G3(x20); (**s**) positive *HOXD13* nuclear and cytoplasm expression in NETG1 (×20); (**t**) positive *HOXD13* nuclear expression in NETG2 (×20); (**u**) positive *HOXD13* nuclear and cytoplasm expression in NETG3 (×20); (**v**) negative *HOXD13* expression in NECG3 (×20).

The immunohistochemical analysis mainly revealed a nuclear localization of *HOXA13*, *HOXB13*, *HOXC13* and *HOXD13* proteins, whereas a cytoplasmic localization was observed only in some areas (Figure 1).

The performed statistical analysis reveals for both *HOXA13*, *HOXC13*, *HOXD13* a strong association with tumor grading classification (Table 2).

**Figure 2:**
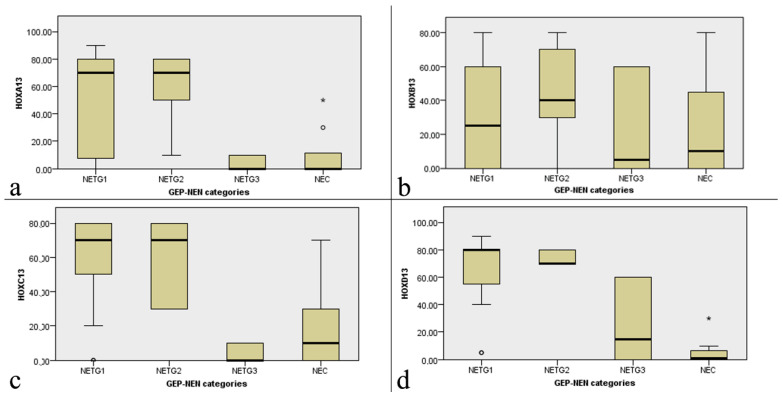
Box plot representation of the relation between paralogous 13 HOX genes expression and GEP- NEN categories (NETG1, NETG2, NETG3, NECG3): (**a**) *HOXA13*; (**b**) *HOXB13*; (**c**) *HOXC13*; (**d**) *HOXD13*. Each value that deviates from the central trend of the distribution is represented in the graph with the symbol °/*.

The general trend of all paralogous 13 HOX genes is a downregulation in high grade tumors. The statistical analysis showed that for *HOXA13* the expression is significantly downregulated in both NETG3 and NEC G3 (Figure 1e–h; Figure 2a). *HOXB13* is expressed heterogeneously in the 4 categories even if with a faint decrease in the G3 categories (Figure 1i–n; Figure 2b). *HOXC13* is overexpressed in NET G1 and NET G2, downregulated in NEC G3, strongly downregulated in NET G3 (Figure 1o–r; Figure 2c). Similarly, *HOXD13* is overexpressed in NET G1 and NET G2 tumors, but downregulated in NET G3, and strongly down regulated in NEC G3 categories (Figure 1s–v; Figure 2d).

### 2.3. HOTAIR Relationship with Tumor Location and GEP-NEN Categories

**Figure 3:**
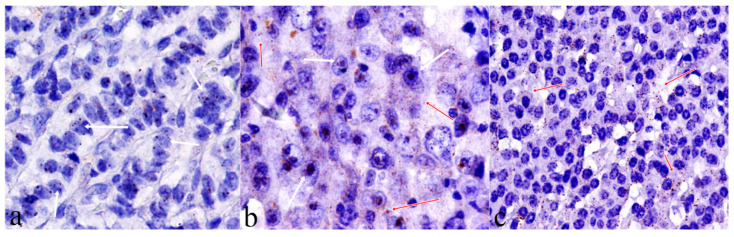
*HOTAIR* ISH signals localization: (**a**) *HOTAIR* nuclear mRNA expression with 4–5 dots/cell in neoplastic cells (40× magnification); (**b**) *HOTAIR* nuclear and cytoplasmic mRNA cluster expression in neoplastic cells (40× magnification); (**c**) *HOTAIR* cytoplasmic mRNA expression, >4 dots/cell in neoplastic cells (40× magnification). White arrows showing nuclear expression while red arrows showing cytoplasmic expression.

In situ hybridization (ISH) *HOTAIR* expression showed a tissue-specific distribution in our cases and no signals in non tumor and stromal cells were detected (Supplementary Figure S1). *HOTAIR* staining can be detected in both nucleus and cytoplasm of tumor cells (Figure 3).

**Figure 4:**
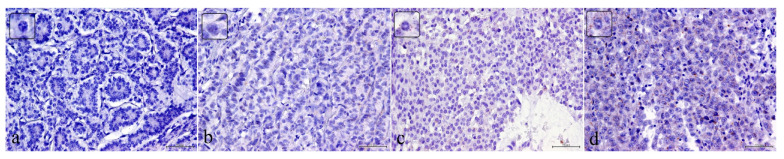
HOTAIR expression in GEP NEN categories: (**a**) Low Score *HOTAIR* mRNA expression in NETG1 cells (40× magnification); (**b**) Intermediate Score *HOTAIR* mRNA expression in NETG2 cells (40× magnification); (**c**) High Score *HOTAIR* mRNA expression in NETG3 cells (40× magnification); (**d**) High Score *HOTAIR* mRNA expression in NECG3 cells (40× magnification).

Statistical elaboration showed that, while there is not direct relation between *HOTAIR* expression and GEP-NEN tumor location, a positive relationship between *HOTAIR* expression and grade has been detected. The *HOTAIR* expression significantly increased with the grade (*p* value = <0.001) (Table 3) (Figure 4).

### 2.4. Relationship Between Paralogous 13 HOX Genes and HOTAIR

**Figure 5:**
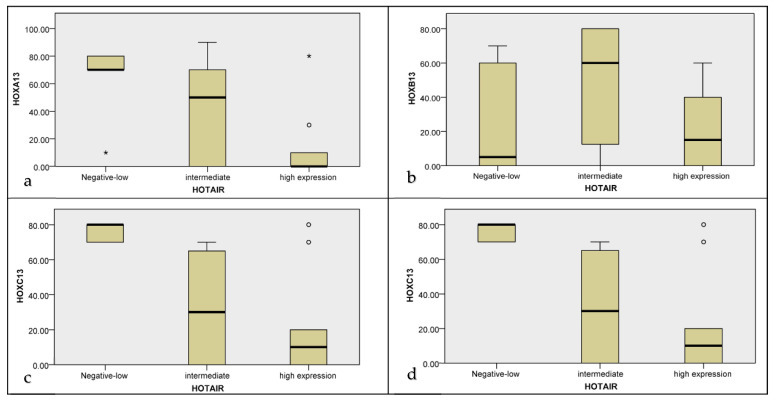
Box plot representation of the relation between HOTAIR expression scores and paralogous 13 HOX proteins expression: (**a**) *HOXA13*; (**b**) *HOXB13*; (**c**) *HOXC13*; (**d**) *HOXD13*. Each value that deviates from the central trend of the distribution is represented in the graph with the symbol °/*.

*HOXA13* is overexpressed in cases with low and intermediate *HOTAIR* scores, while it is almost completely absent in cases with high *HOTAIR* scores (*p* value = 0.004) (Figure 5a). No relationship between *HOXB13* and *HOTAIR* has been detected (*p* value = 0.118) (Figure 5b).

*HOXC13* is overexpressed in cases with low *HOTAIR* scores, and de-regulated in cases with high *HOTAIR* scores (*p* value = 0.001) (Figure 5c). *HOXD13* is upregulated in cases with low *HOTAIR* scores, while it is downregulated in cases with intermediate/high *HOTAIR* scores (*p* value = 0.001) (Figure 5d).

## 3. Discussion

The incidence of GEP-NENs has increased over the last few decades. About 2/3 of patients can present distant metastases and the five-year survival rate exceeds 60%. For this reason, the incidence of GEP-NEN is higher than that of pancreatic, gastric and oesophageal adenocarcinomas, making it the second most prevalent type of cancer of the gastrointestinal tract [1]. Moreover, the recent classification criteria based on tumor grading and cell differentiation, has allowed the identification of different categories for GEP-NENs, with very different prognostic implications [3,4]. Consequently, the possibility of early detection and the development of appropriate therapeutic strategies promoted the research of new diagnostic and prognostic biomarkers.

In this study we focused the attention on the role of HOX13 paralogous genes in GEP-NEN tumors and investigated the potential contribute of the lncRNA *HOTAIR* in the definition of their prognostic categories.

The alteration of expression of the HOX genes seems to be strongly associated with the different development sites of GEP-NEN tumors but also with the 4 grading related categories. In detail, *HOXA13* always appears to be downregulated in the prognostically most unfavorable category, NECG3, compared to the other tumor types. Similarly, *HOXC13* and *HOXD13* are downregulated in NECG3.

During development, paralogous 13 HOX genes are involved in mediating the transition from the early to the late-distal limb program, controlling the spatial-temporal expression patterns of target genes [20] and in mediating gut and urogenital system formation [21,22,23]. However, many of them are still active in adult human organs and tissues and frequently deregulated in human cancers [8,11,23]. We have recently shown their de-regulation in colon cancer, describing the absence of expression of *HOXA13*, *HOXB13*, *HOXC13* and *HOXD13* in the normal colon mucosa, a slight increase in expression in the transitional mucosa, up to in many cases over-expressed in the tumor. In particular, HOX *B13* and *HOXC13* expression strongly correlated with lymph nodes metastasis and showed a prevalent expression in CRC samples with a very poor prognosis. On the contrary, in pancreatic cancer, *HOXD13* displayed on opposite trend being strongly down-regulated in cancer cells. For this reason, for these genes a dual role has been suggested during tumor evolution, as oncogenes and tumor suppressor genes [9].

The ability of the HOX genes to molecularly characterize and differentiate the categories of GEP-NEN at risk of progression could add an important step to the understanding of the molecular mechanisms associated with the evolution of this tumor.

Although the entire HOX network plays a central role in cancer development and progression, the most posterior genes of the network are crucial in modulating these processes, in cooperation with co-localizing lncRNAs [24].

In particular, the lncRNA HOTAIR is able to act as a regulator of chromatin states by binding PRC2, with its 5′end and at 3′ end, with LSD1 (lysine-specific demethylase 1) promoting the epigenetic activation/repression of gene expression [25].

In this study we have analyzed the expression of HOTAIR on our GEP-NEN cases, highlighting its gradual upregulation from the NETG1 to the more prognostically unfavorable NECG3 tumor type.

Currently, HOTAIR is considered as an important biomarker associated with the pathogenesis and progression of many tumors [26,27]. In recent years, its prognostic role has appeared even more evident being involved also in the main mechanisms of therapeutic resistance [28]. In different solid tumors, its role as a circulating marker has been shown, suggesting a main role in monitoring tumor evolution and response to specific therapies [29].

Our data support the idea that *HOTAIR*, as well as being an important prognostic marker, may represent also a potential therapeutic target in GEP-NEN tumors. In fact, different studies showed that the direct or indirect block/inhibition of HOTAIR may represent a new and effective cancer therapeutic strategy. The majority of the functional studies on *HOTAIR* performed its direct inhibition by siRNA methods, but the translation of these methods in clinical practice is complicated. Instead, more recently the design of small molecules able to specifically interfere with conserved RNA structures and to block HOTAIR protein complexes have proved more useful [30,31].

In the present study we also tried to establish a relationship between the expression of the paralogous 13 HOX genes and *HOTAIR* in GEP/NEN categories. *HOXA13*, *HOXC13* and *HOXD13* expression appears to be inversely related with *HOTAIR* expression, highlighting an inverse relationship with the prognostically unfavourable grading category.

The data appears quite in line with what is described in the literature, in particular for *HOXD13*. In fact, it is known that mechanistically HOTAIR is responsible for gene-silencing of an entire area of chromosome 12 including the HOXD locus and a series of metastasis suppressor genes, promoting metastatic switch [32]. The only major limitation of this study is the low availability of recruitable patients, being very rare diseases and often treated in different Institutions.

In conclusion, although an accurate molecular characterization of GEP-NENs is necessary, especially to predict their evolution, our data could suggest paralogous 13 HOX genes expression combined with lncRNA *HOTAIR* as a new useful tool for the prognostic definition and management of GEP-NENs patients.

## 4. Materials and Methods

### 4.1. Patients and Specimens

Thirty-four GEP-NEN patients admitted to the National Cancer Institute “Giovanni Pascale” of Naples, between 2015 and 2019, were recruited in this study. All patients had provided written informed consent for the use of tumor samples according to the institutional regulations and the study was approved by the ethics committee of the National Cancer Institute “G. Pascale”(Project name: “Fighting Cancer resistance: Multidisciplinary integrated Platform for a technological Innovative Approach to Oncotherapies (Campania Oncotherapies)”- Ethical approval n.40/19 OSS- Date 21 November 2019). All cases have been reviewed by two pathologists (FT, MDB) and graded and staged according to WHO 2017 and 2019 classification criteria (NET-G1, NET-G2, NET-G3, NEC-G3) on tissue sections. The 4 main categories are distinguished on the basis of the proliferative activity, measured through the mitotic count and the Ki67 expression. Medical records have been reviewed for clinical information, including histologic parameters, assessed on standard H&E-stained slides combined with immunohistochemical staining with neuroendocrine markers (Cromogranin, Synaptophisin, CD56), and tumor location.

### 4.2. Immunohistochemistry Analysis

All selected samples derived from formalin-fixed, paraffin embedded tissues (FFPE). Paraffin slides were then deparaffinized in xylene and rehydrated through graded alcohols. Antigen retrieval was performed with slides heated in 0.0.1 M citrate buffer (pH 6.0.) for 20 min at 97 °C. After antigen retrieval, the slides were allowed to cool. The slides were rinsed with TBS and the endogenous peroxidase has inactivated with 3% hydrogen peroxide. After protein block (BSA 5% in PBS 1×), the slides were incubated with primary antibody to human *HOXA13* (dilution 1:200, cod. Ab106503, Abcam, Cambridge, UK), *HOXB13* (dilution 1:300, cod. ab28575, Abcam, Cambridge, UK), *HOXC13* (dilution 1:1200, cod.ab55251, Abcam, Cambridge, UK), *HOXD13* (dilution 1:100, cod. Ab19866, Abcam, Cambridge, UK) overnight. Sections were incubated with mouse anti-rabbit or goat anti-mouse secondary IgG biotinylated secondary antibody for 30 min. Immunoreactivity was visualized by means of avidin–biotin–peroxydase complex kit reagents (Novocastra, Newcastle, UK) as the chromogenic substrate. Finally, sections were weakly counterstained with haematoxylin and mounted.

### 4.3. Evaluation of Immunostaining

Antigen expression was independently evaluated by two experienced pathologists (FT/MDB) using light microscopy. For paralogous 13 HOX genes nuclear and cytoplasmic localization were considered. All values of immunostaining were expressed only in percentage terms of positive cells. The percentage of positive cancer cells was evaluated in each sample by counting the number of positive cells over the total cancer cells in 10 non-overlapping fields using ×400 magnification as previously described [15].

### 4.4. RNA In Situ Hybridization Assay (RNA ISH)

In situ detection of HOTAIR was performed using the RNAscope (RNAscope^®^ 2.5 HD Detection Reagent-BROWN User Manual) according to the manufacturer’s instructions. The tissue sections were boiled at 95 °C for 30 min in Target Retrieval solution. Protease treatment was then applied at 40 °C for 40 min. Then, we hybridized the Hotair probe for 2 h at 40 °C. The detection kit (BROWN) was used to amplify and reveal the signal, according to the manufacturer’s instructions. The assay was performed using two controls: peptidylprolyl isomerase B (cyclophilin B) (PPIB) mRNA, a positive control and duplex negative control probe (dapB), a negative control. The slides were independently evaluated by two separate observers (FT/MDB). Positive staining was indicated by signals as brown dots present in the nucleus and/or cytoplasm. The number of signal staining was counted in 60 tumor cells. *HOTAIR* has an expression level varying between 0 to >10 copies per cell. We used a semi-quantitative scoring utilizing the estimated number of dots present within each cell boundary. We have categorized staining into 3 scores: High Score (>6 dots/cell- clusters), Intermediate Score (>3 <6 dots/cell), Low Score (no staining or <3 dot/cell) (Figure 1).

### 4.5. Statistical Analysis

Non-parametric tests were used to compare independent groups of numerical data. Differences in the expression of *HOXA13*, *HOXB13*, *HOXC13*, *HOXD13* according to age, gender, location and GEP-NEN categories were analyzed using Mann–Whitney U-test and Kruscall-Wallis tests The Pearson χ2 test was used in order to determine relation between Hotair expression and the variables included in the study. *p* < 0.05 was considered statistically significant. All tests used were two-tailed. All statistical analyses were carried out using SPSS version 20.0 software (SPSS, Chicago, IL, USA). Results are illustrated by boxplot graphs, a standardized way of displaying the distribution of data based on a five number summary (“minimum”, first quartile (Q1), median, third quartile (Q3), and “maximum”).

## Figures and Tables

**Table 1:** Clinic-pathological characteristics of GEP-NEN patients.

Age	<65 years	16 (47.1)
	≥65 years	18 (52.9)
Gender	M	17 (50)
	F	17 (50)
GEP-NEN categories	NET G1	14 (41.2)
	NET G2	7 (20.6)
	NET G3	3 (8.8)
	NEC G3	10 (29.4)

**Table 2:** Relation between *HOXA13*, *HOXB13*, *HOXC13*, *HOXD13* and tumor grading classification.

	Age	Gender	GEP-NEN Categories
*HOXA13*	0.154	0.586	0.002
*HOXB13*	0.621	0.892	0.627
*HOXC13*	0.102	0.838	0.002
*HOXD13*	0.109	0.708	<0.001

**Table 3:** Relation between lncRNA *HOTAIR* and tumor grading classification.

HOTAIR Expression		Negative-Low	Intermediate	High	*p* Value
Age	<65 years	5 (17.2)	5 (17.2)	3 (10.3)	0.449
	≥65 years	4 (13.8)	4 (13.8)	8 (27.6)	
Gender	M	6 (20.7)	3 (10.3)	7 (24.1)	0.411
	F	3 (10.3)	6 (20.7)	4 (13.8)	
GEP-NEN categories	NETG1	9 (31)	2 (6.9)	1 (3.4)	<0.001
	NETG2	0	3 (10.3)	1 (3.4)	
	NETG3	0	2 (6.9)	1 (3.4)	
	NECG3	0	2 (6.9)	8 (27.6)	

## Supplementary Materials

The following are available online at https://www.mdpi.com/article/10.3390/ijms22137049/s1.

## Abbreviations

GEP-NENs	Gastro-Entero-Pancreatic Neuroendocrine Neoplasms
GEP-NETs	Gastro-Entero-Pancreatic Neuroendocrine Tumors
GEP-NECs	Gastro-Entero-Pancreatic Neuroendocrine Carcinomas
WHO	World Health Organization
HOX	Homeobox
LncRNAs	Long non coding RNAs
HOTAIR	Hox transcript antisense intergenic RNA
NENs	Neuroendocrine neoplasms
CRC	Colorectal cancer
ISH	In Situ Hybridization
