# Supplementary material for: Aberrant Expression of Long Non Coding RNA HOTAIR and De-Regulation of the Paralogous 13 HOX Genes Are Strongly Associated with Aggressive Behavior of Gastro-Entero-Pancreatic Neuroendocrine Tumors
Source: Int J Mol Sci. 2021 Jun 30;22(13):7049. doi: 10.3390/ijms22137049 (PMC8268982; doi:10.3390/ijms22137049)
Supplement: Supplementary file 1 [file ijms-22-07049-s001.zip › ijms-1268473-supplementary.pdf]

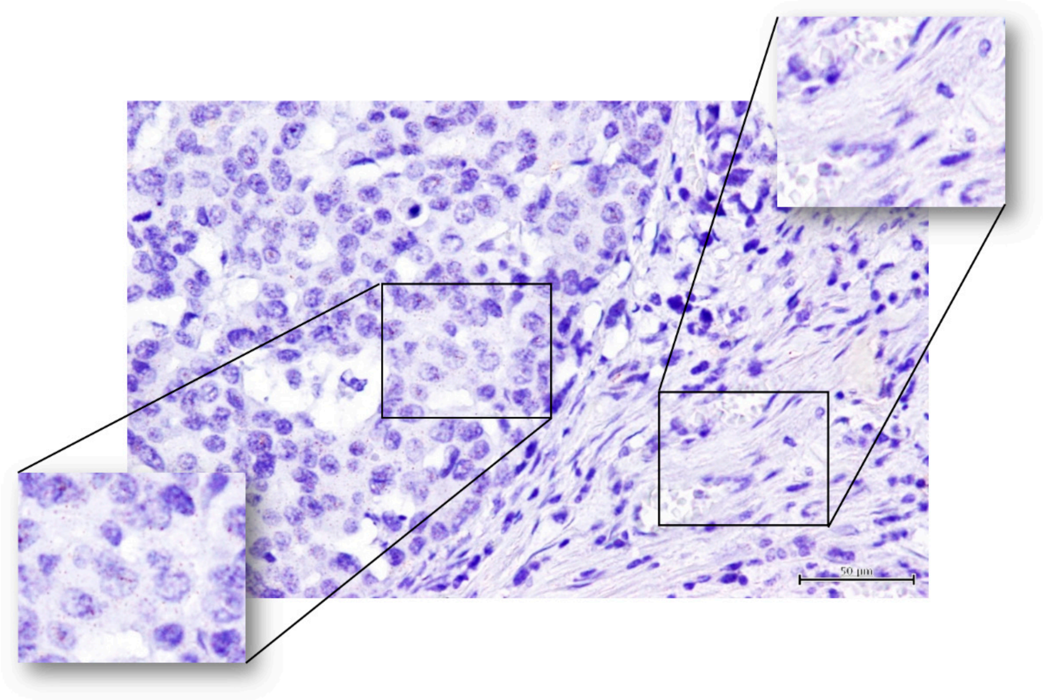

Supplementary Figure S1. HOTAIR expression in a GEP NEN sample. On the left details of the positive staining in tumor cells and on the right details of the negative staining in stromal cells (40X).
